# Serum amyloid P component and pro-platelet basic protein in extracellular vesicles or serum are novel markers of liver fibrosis in chronic hepatitis C patients

**DOI:** 10.1371/journal.pone.0271020

**Published:** 2022-07-07

**Authors:** Kumiko Shirai, Hayato Hikita, Sadatsugu Sakane, Ryohei Narumi, Jun Adachi, Akira Doi, Satoshi Tanaka, Yuki Tahata, Ryoko Yamada, Takahiro Kodama, Ryotaro Sakamori, Tomohide Tatsumi, Eiji Mita, Takeshi Tomonaga, Tetsuo Takehara

**Affiliations:** 1 Department of Gastroenterology and Hepatology, Osaka University Graduate School of Medicine, Suita, Osaka, Japan; 2 Laboratory of Proteome Research/Proteome for Drug Discovery, National Institute of Biomedical Innovation, Health and Nutrition, Ibaraki, Osaka, Japan; 3 Department of Gastroenterology and Hepatology, National Hospital Organization Osaka National Hospital, Chuoku, Osaka, Japan; Pacific Northwest National Laboratory, UNITED STATES

## Abstract

Extracellular vesicles (EVs) contain proteins, mRNAs, and microRNAs, and their cargos have emerged as novel diagnostic markers in various diseases. We aimed to discover novel and noninvasive biomarkers of liver fibrosis by proteomic analysis using serum EVs in patients with chronic hepatitis C. We performed shotgun proteomics using serum EVs isolated from 54 patients with histologically assessed liver fibrosis. Shotgun proteomics identified a total of 974 proteins, and 445 proteins were detected in more than half of the patients. Among them, a total of 9 proteins were identified as proteins that tended to increase or decrease with liver fibrosis with a significance of p<0.005 and that were different between F1-2 patients and F3-4 patients with a significance of p<0.01. Among the 9 proteins, targeted proteomics using serum EVs isolated from the sera of another 80 patients with histologically assessed liver fibrosis verified that serum amyloid P component (SAP) and pro-platelet basic protein (PPBP) levels in EVs significantly decreased with the progression of liver fibrosis and were significantly lower in F3-4 patients than in F1-2 patients. The diagnostic accuracies of SAP and PPBP in EVs for the liver fibrosis stage were comparable to those of type IV collagen 7S, hyaluronic acid, and the fibrosis-4 index (FIB-4 index). Moreover, serum SAP and PPBP levels correlated with the levels in EVs, and the ability of serum SAP and PPBP to diagnose liver fibrosis stage was also comparable to the abilities of type IV collagen 7S, hyaluronic acid, and the FIB-4 index. In conclusion, proteomic analysis of serum EVs identified SAP and PPBP as candidate biomarkers for predicting liver fibrosis in patients with chronic hepatitis C. In addition, SAP and PPBP levels in serum are strongly correlated with those in EVs and could represent markers of liver fibrosis.

## Introduction

Liver fibrosis is caused by a variety of chronic liver diseases, such as viral hepatitis, alcoholic liver disease, and nonalcoholic fatty liver disease [1–4]. Continuous progression of fibrosis leads to cirrhosis and liver failure. As patients with liver cirrhosis often have complications such as ascites, gastroesophageal varices, encephalopathy and hepatocellular carcinoma, they need to be followed up carefully [5]. Therefore, it is important to accurately evaluate the liver fibrosis stage in patients with chronic liver diseases. Liver biopsy is the gold standard for the assessment of liver fibrosis, but it is an invasive method [6]. In addition, there are concerns with liver biopsy, such as sampling error and observer variability [7]. Although other diagnostic methods, such as type IV collagen 7S, hyaluronic acid, the fibrosis-4 index (FIB-4 index), elastography with magnetic resonance imaging and ultrasonography, are reported as noninvasive fibrosis markers [8–11], other accurate and noninvasive methods for evaluating liver fibrosis in patients with chronic liver diseases are still needed.

Extracellular vesicles (EVs) are nanosized, membrane-bound vesicles that are secreted from various types of human cells and circulate in body fluids, such as blood, urine, and cerebrospinal fluid [12]. EVs contain proteins, microRNAs, and mRNAs and play a pivotal role in intercellular communication [13]. The cargos of EVs are considered specific to their secretory cells and environment, and they are stable in most body fluids due to protection by lipid bilayers. Thus, EVs are expected to be promising resources for the discovery of new biomarkers and therapeutic targets without the influence of abundant serum proteins. Proteins in EVs have been reported to be potential novel biomarkers in various diseases [14–16], but there are few reports with respect to liver fibrosis. In the present study, we aimed to discover novel biomarkers of liver fibrosis using EV proteins in patients with chronic hepatitis C.

## Materials and methods

### Patients with chronic hepatitis C

A total of 418 patients with chronic hepatitis C received direct-acting antiviral (DAA) treatment at Osaka University Hospital from September 2014 to June 2019. Among them, we excluded patients who had not undergone liver biopsy or could not be assessed for liver fibrosis, those with a previous history of hepatocellular carcinoma, those with a previous history of liver transplantation and those with other liver diseases (coinfection of hepatitis B virus and autoimmune hepatitis). Among the 263 patients remaining after exclusion, we enrolled 54 patients in a trial cohort who received sofosbuvir/ledipasvir treatment from September 2015 to August 2016, subsequently achieved a sustained virologic response, received follow up over 48 weeks after the end of treatment, and who were 40–80 years old without previous hepatitis B virus infection and no history of malignancy in other organs (S1 Fig, S1 Table). As a validation cohort, we randomly selected 20 patients with each fibrosis stage who achieved a sustained virologic response from the remaining 209 patients (S2 Fig, S2 Table). These studies were performed in line with the ethics guidelines outlined in the Declaration of Helsinki and were approved by the Institutional Research Board of Osaka University Hospital (No. 17302). All patients signed written informed consent forms before participating in the study.

### Patients with chronic hepatitis B, nonalcoholic fatty liver disease (NAFLD) and colorectal polyps

For patients with chronic hepatitis B before treatment with nucleos(t)ide analogs and NAFLD, we used stocked sera from patients whose fibrosis stage was assessed by liver biopsy at Osaka University Hospital and National Hospital Organization Osaka National Hospital. As controls, we used sera from patients with colorectal polyps who were 40–50 years old without liver disease complications at Osaka University Hospital. These studies were performed according to the ethics guidelines outlined in the Declaration of Helsinki and were approved by the Institutional Research Board of Osaka University Hospital (No. 17302) and National Hospital Organization Osaka National Hospital (No. 20124).

### Histological assessment of fibrosis

Liver biopsy was performed using a 16-G biopsy needle. Liver pathologists assessed liver biopsy specimens and scored the stage of fibrosis by referring to the METAVIR score [17]. For patients with NAFLD, liver fibrosis was scored based on the Kleiner classification [18].

### Isolation and solubilization of extracellular vesicles from patient sera

Sera were stored at -80°C until analysis. A total of 200 μl of serum was diluted with 300 μl of PBS and centrifuged at 4,000×g for 3 min to remove large debris. Then, the supernatants were passed through a 0.22 μm spin filter (catalog number: 5185–5990, Agilent Technologies, Santa Clara, CA). Phosphatidylserine-positive extracellular vesicles (EVs) were isolated by an affinity-based method [19] using the Magcapture^™^ Exosome Isolation Kit PS (catalog number: 293–77601, FUJIFILM Wako Pure Chemical Corporation, Osaka, Japan) according to the manufacturer’s procedure. Isolated EVs were solubilized in phase-transfer surfactant buffer composed of 50 mM NH_4_HCO_3_, 12 mM sodium deoxycholate, and 12 mM sodium N-lauroyl sarcosinate [20] and incubated at 95°C for 5 min. Then, the samples were sonicated by using a BIORUPTOR II (Sonicbio. Co., Ltd. Kanagawa, Japan) for 10 min. After sonication, the samples were centrifuged at 20,000×g for 10 min, and the supernatants were collected.

### Digestion of the protein in EVs for proteome analysis

The solubilized proteins were reduced with 10 mM tris (2-carboxyethyl) phosphine hydrochloride for 1 hour at 37°C, alkylated with 20 mM iodoacetamide for 30 min at 37°C and then quenched by 21 mM L-cysteine. Next, samples were digested with 2 mAU lysyl endopeptidase (Wako-Chemical, Tokyo, Japan) and 1 μg of trypsin (Proteomics grade, Roche, Mannheim, Germany) at 37°C overnight. The digested solutions were acidified with 1% trifluoroacetic acid and centrifuged at 20,000×g for 10 min to remove the detergents. For nontargeted proteomics, the supernatant was desalted using StageTips as previously described [21]. It was dried using a SpeedVac and then stored at -80°C until isobaric chemical labeling. For targeted proteomics, stable isotope-labeled peptide standards were added to the supernatant. This was followed by clean-up using C18-SCX StageTip, as previously described [22]. The samples were stored at -80°C until they were analyzed by mass spectrometry.

### Nontargeted proteomics: Shotgun proteomics

The desalted peptides for nontargeted proteomics were labeled with reagents for isobaric peptide labeling with the Tandem Mass Tag (TMT)-10 plex (Thermo Fisher Scientific, Bremen, Germany) according to the manufacturer’s protocol.

The TMT-labeled sample mixtures were separated into seven fractions by using C18-SCX StageTip [22], and then the fractions were stored at -80°C until mass spectrometry analysis. The TMT-labeled peptides were analyzed by a Q-Exactive plus mass spectrometer (Thermo Scientific, Bremen, Germany) with an UltiMate 3000 Nanoflow high-performance LC system (Dionex, Sunnyvale, CA) and an HTC-PAL autosampler (CTC Analytics, Zwingen, Switzerland). Raw files were searched with MaxQuant (version 1.5.7.0) [23] against the UniProt Human Protein Database (FASTA version 2017–01). The precursor mass tolerance was set to 7 ppm, and the fragment ion mass tolerance was set to 0.01 Da. Peptides and proteins were accepted with a false discovery rate of < 1%, which was estimated based on the number of accepted hits from the reverse database. The detailed protocol of nontargeted proteomics is described in S1 File.

### Selection of target peptides and synthetic peptides

Target peptides were selected from those identified in the shotgun proteomic analysis according to the following rules: 1) peptides with sequences that were not shared between multiple genes, 2) peptides that were completely cleaved and had no methionine, and 3) peptides with lengths of less than 20 amino acids because of the higher sensitivity of the selected reaction monitoring (SRM) analysis.

Stable synthetic isotope-labeled peptides with C-terminal 15N- and 13C-labeled arginine or lysine residues (isotopic purity > 99%) were purchased from JPT Peptide Technologies Gmbh (Berlin, Germany).

### Targeted proteomics: Selected reaction monitoring

The digested peptides containing stable isotope-labeled peptide standards were analyzed by using a TSQ-Vantage triple quadruple mass spectrometer (Thermo Fisher Scientific, Bremen, Germany) with a nano-LC interface (AMR, Tokyo, Japan), Paradigm MS2 (Michrom BioResources, Auburn, CA), and an HTC-PAL autosampler (CTC Analytics, Zwingen, Switzerland). The raw files acquired in targeted proteomics were analyzed using Skyline software [24]. SRM signal peaks corresponding to each target peptide were assigned by comparison with an isotope-labeled peptide internal standard of each counterpart. The quantitative values of the target peptides were obtained as ratios of the endogenous target peptides to the stable isotope-labeled peptide internal standard using 1 transition per peptide with the highest signal. The detailed protocol of targeted proteomics is described in S1 File.

### Enzyme-linked immunosorbent assay

Quantitative evaluation of serum amyloid P component (SAP) and pro-platelet basic protein (PPBP) in serum was performed by using commercially available sandwich enzyme-linked immunosorbent assay (ELISA) kits, namely, Human APCS ELISA Kit (catalog number; KE00122, Proteintech, Chicago, IL) and Human CXCL7/PBP ELISA Kit (catalog number; ab216171 Abcam, Cambridge, UK). For the measurement of SAP, serum samples were diluted 1:200,000 with the supplied dilution buffer. For the measurement of PPBP, serum samples were diluted 1:128,000 with the supplied dilution buffer. The procedure was performed according to each manufacturer’s protocol.

### Data analysis and statistics

Data are expressed as the mean ± standard deviation. Fisher’s exact test was used to analyze categorical data. The Kruskal–Wallis test was performed for multiple comparisons. Statistical analyses were performed with Mann–Whitney U tests to assess differences between unpaired groups and the Wilcoxon Signed-rank sum test for paired groups. The Jonckheere-Terpstra test was used to assess the trend with liver fibrosis stage. Correlations were evaluated using the Pearson product-moment correlation coefficient. To assess the diagnostic performance of the identified proteins, receiver operating characteristic curve analysis was performed, and the area under the curve (AUC) was used to evaluate the predictive power. The AUCs were compared by using the Delong test. A *P* value < 0.05 was considered to indicate statistical significance unless otherwise indicated. All analyses were performed using R (version 4.1.0) and R Studio (version 1.4.1717) software and Prism v.9.2.0 for Windows.

The heatmap was illustrated using the ComplexHeatmap (version 2.8.0) package.

Other diagrams were produced using Prism v.9.2.0 for Windows. Fisher’s exact test, Mann–Whitney U test, and Kruskal–Wallis test were performed by using the Rcmdr (version 2.7–1) package or the BiocManager (version 1.30.16) package. Delong tests were performed by using the pROC (version 1.17.0.1) package. The Jonckheere-Terpstra trend test was performed by using the clinfun (version 1.0.15) package. Other statistical analyses were performed utilizing Prism v.9.2.0 for Windows.

## Results

### Shotgun proteomic analysis of proteins in serum EVs

**Fig 1:**
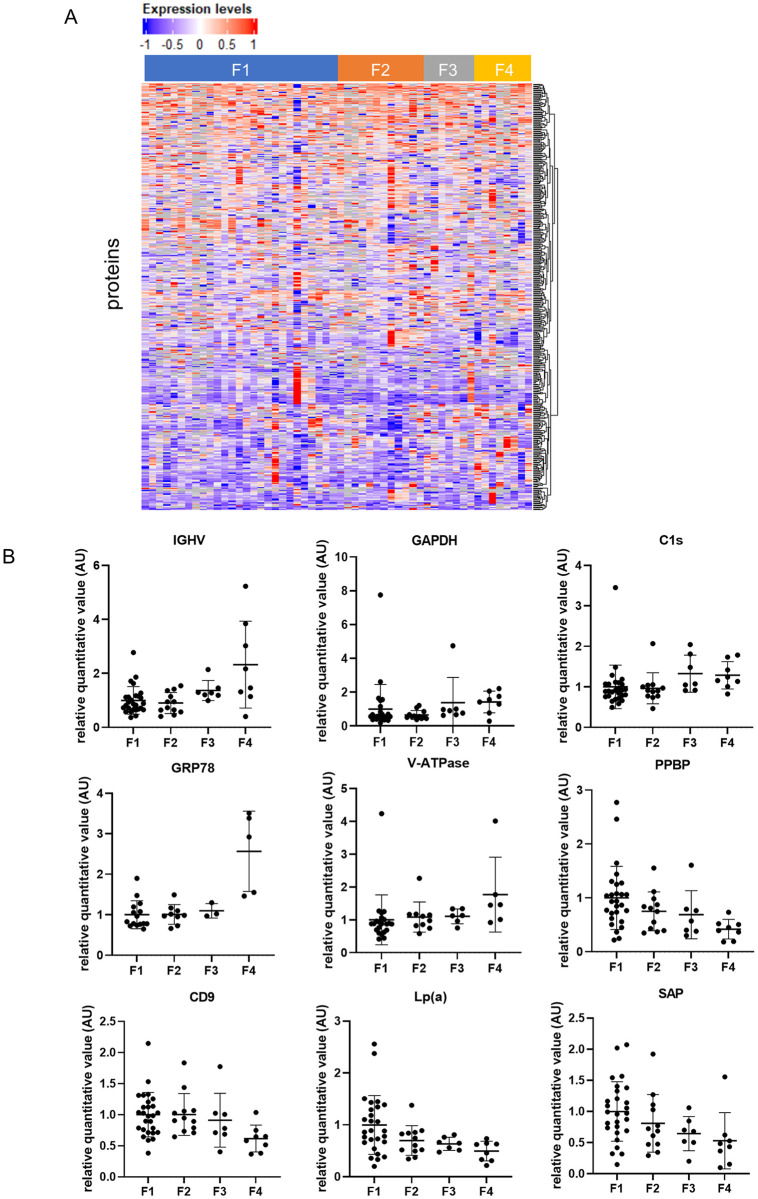
The results of shotgun proteomic analysis. (A) Heatmap of 445 EV proteins that were detected in more than half of the patients. (B) Relative quantitative value of IGHV, GAPDH, C1s, GRP78, V-ATPase, PPBP, CD9, Lp(a) and SAP. The dot plot shows individual values. The data are expressed as the mean ± standard deviation. Abbreviations: IGHV, Ig heavy chain V-II region WAH; GAPDH, glyceraldehyde-3-phosphate dehydrogenase; C1s, complement C1s subcomponent; GRP78, 78 kDa glucose-regulated protein; V-ATPase, ATPase H+ transporting V0 subunit a1; PPBP, pro-platelet basic protein; CD9, CD9 antigen; Lp (a), lipoprotein (a); SAP, serum amyloid P component.

We performed a shotgun proteomic analysis by the TMT method using EVs isolated from the sera of 54 patients with chronic hepatitis C before DAA treatment in the trial cohort (S1 Table). The shotgun proteomic analysis detected 974 proteins, and 445 proteins were detected in more than half of the patients (Fig 1A). Type IV collagen 7S, a known fibrosis marker, was not present in the 974 proteins. We selected proteins that tended to increase or decrease with liver fibrosis with a significance of p<0.005 by the Jonckheere-Terpstra trend test and whose expression levels were different with a significance of p<0.01 between patients with F1-2 and those with F3-4. Among 445 proteins, 9 proteins were identified as proteins whose expression levels significantly tended to increase, and 7 proteins were identified as proteins whose expression levels significantly tended to decrease with the progression of fibrosis (Table 1). Among these 16 proteins, Ig heavy chain V-II region WAH (IGHV), glyceraldehyde-3-phosphate dehydrogenase (GAPDH), complement C1s subcomponent (C1s), 78 kDa glucose-regulated protein (GRP78), ATPase H+ transporting V0 subunit a1 (V-ATPase), pro-platelet basic protein (PPBP), CD9 antigen (CD9), lipoprotein (a) (Lp(a)) and serum amyloid P component (SAP) were significantly different between patients with F1-2 and those with F3-4 (Table 1). Along with the progression of liver fibrosis, IGHV, GAPDH, C1s, GRP78 and V-ATPase levels significantly increased, and PPBP, CD9, Lp(a) and SAP levels significantly decreased (Fig 1B).

**Table 1:** List of the proteins that increased or decreased significantly with the progression of liver fibrosis.

Protein names	P value calculated by Jonckheere-Terpstra test	F3-4 vs. F1-2 Log2 fold change	F3-4 vs. F1-2 univariate p value
Ig heavy chain V-II region WAH (IGHV)	0.0021	0.60	0.00032
glyceraldehyde-3-phosphate dehydrogenase (GAPDH)	0.0026	0.36	0.00054
complement C1s subcomponent (C1s)	0.0025	0.85	0.0012
78 kDa glucose-regulated protein (GRP78)	0.0016	0.64	0.0017
ATPase H+ transporting V0 subunit a1 (V-ATPase)	0.0010	0.48	0.0048
14-3-3 protein gamma	0.0023	0.54	0.020
chloride intracellular channel protein 1	0.0045	0.30	0.021
calreticulin	0.00047	0.91	0.038
oncoprotein-induced transcript 3 protein	0.0028	0.29	0.11
pro-platelet basic protein (PPBP)	0.00035	-0.91	0.0028
CD9 antigen (CD9)	0.0046	-0.51	0.0049
lipoprotein (a) (Lp(a))	0.00027	-0.40	0.0052
serum amyloid P component (SAP)	0.00082	-0.75	0.0086
haptoglobin	0.00037	-0.39	0.016
beta-2-glycoprotein 1	0.00048	-0.56	0.055
zinc finger protein 648	0.00035	-0.73	0.058

Note: The trend of each protein with the stage of liver fibrosis was assessed by the Jonckheer-Terpstra test. The difference between patients with F1-2 and those with F3-4 for each protein was assessed by the Mann–Whitney U test.

### Verification by target proteomic analysis

**Fig 2:**
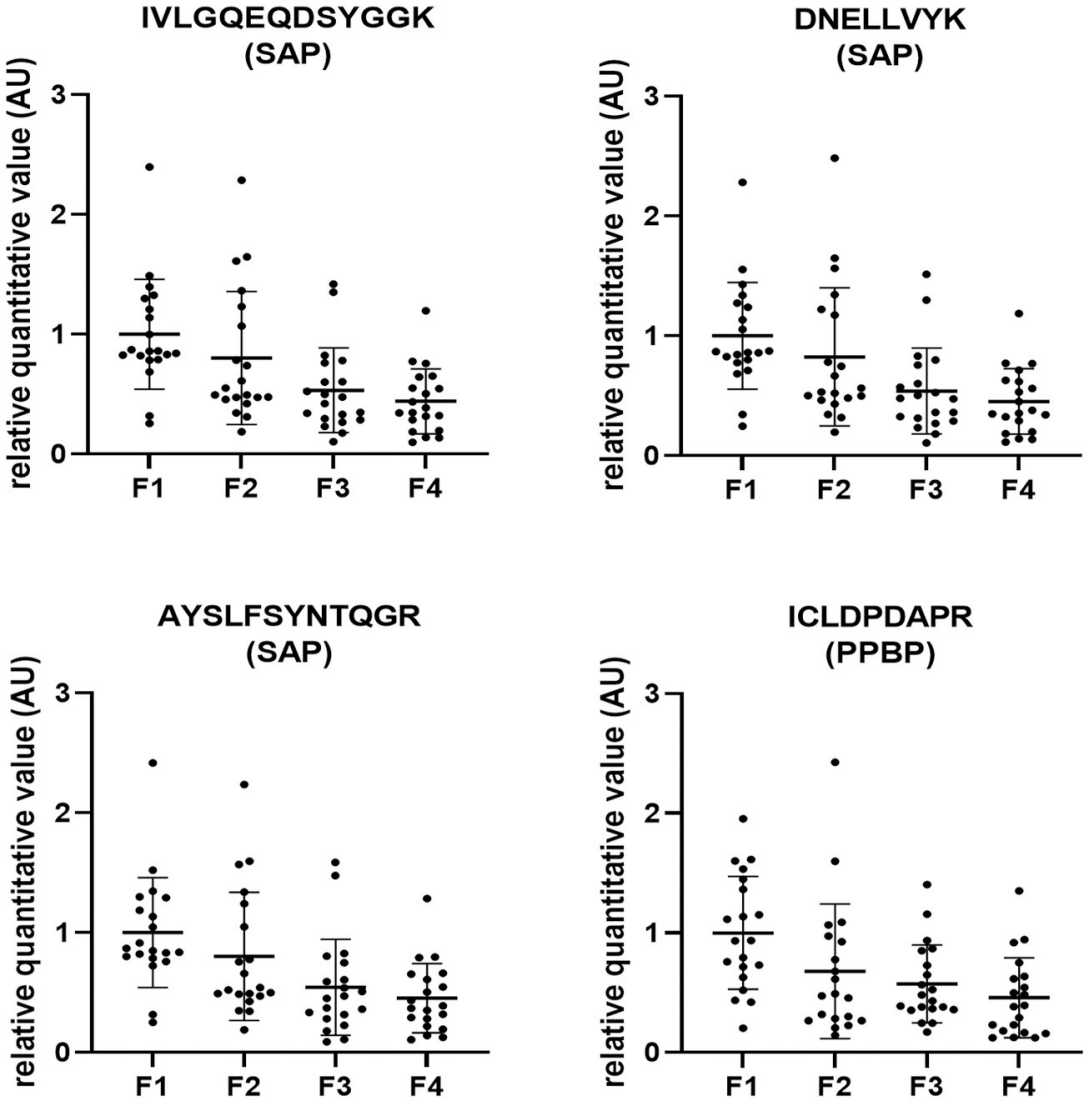
The relative quantitative values of 3 SAP-targeted peptides and 1 PPBP-targeted peptide. The dot plot shows individual values. The data are expressed as the mean ± standard deviation. Abbreviations: SAP, serum amyloid P component; PPBP, pro-platelet basic protein.

To verify the results of the trial study, we examined the levels of the 9 proteins, IGHV, GAPDH, C1s, GRP78, V-ATPase, PPBP, CD9, Lp(a) and SAP, in EVs isolated from the sera of another 80 patients with chronic hepatitis C before DAA treatment (S2 Table). We selected three peptides for each protein and performed targeted proteomic analysis of these peptides by the SRM method. Three peptides are specific for one corresponding protein. Since the sensitivity of each peptide was different, not all three peptides corresponding to each protein were detected in all patients (Table 2). Similar to a trial study, we selected peptides that tended to increase or decrease with liver fibrosis with a significance of p<0.005 by the Jonckheere-Terpstra trend test and whose expression levels were different with a significance of p<0.01 between patients with F1-2 and those with F3-4. Among the 27 target peptides of the 9 proteins, 3 SAP-targeted peptides and 2 PPBP-targeted peptides significantly tended to decrease with liver fibrosis (Table 2). Furthermore, among the 5 peptides, 3 SAP-targeted peptides and 1 PPBP-targeted peptide were significantly different between patients with F1-2 and those with F3-4 (Table 2). All relative quantitative values of the 3 SAP-targeted peptides and 1 PPBP-targeted peptide showed a significantly decreasing trend as liver fibrosis progressed (Fig 2).

**Table 2:** Results of targeted proteomic analysis.

Target protein	Peptide	Number of cases detected	P value calculated by Jonckheere-Terpstra test	F3-4 vs. F1-2 Log2 fold change	F3-4 vs. F1-2 p value
SAP	IVLGQEQDSYGGK	80	1.2E-06	-1.03	1.3E-05
SAP	DNELLVYK	80	1.9E-06	-0.97	1.5E-05
SAP	AYSLFSYNTQGR	80	2.3E-06	-0.98	2.1E-05
PPBP	ICLDPDAPR	80	7.2E-05	-0.86	3.6E-03
PPBP	NIQSLEVIGK	80	1.2E-03	-0.59	0.011
CD9	SCPDAIK	80	0.012	-0.81	0.025
PPBP	EESLDSDLYAELR	54	0.022	-0.69	0.092
Lp (a)	TPAYYPNAGLIK	80	0.044	-0.55	0.084
Lp (a)	YICAEHLAR	80	0.044	-0.74	0.15
C1s	EDTPNSVWEPAK	62	0.053	-0.064	0.74
CD9	EVQEFYK	80	0.056	-0.47	0.062
CD9	DVLETFTVK	80	0.061	-0.50	0.068
Lp (a)	GTYSTTVTGR	80	0.080	-0.35	0.20
GRP78	ITPSYVAFTPEGER	61	0.16	0.019	0.89
IGHV	NQFSLNLR	80	0.34	0.023	0.76
IGHV	VTISVDTSR	80	0.38	-0.19	0.62
C1s	IIGGSDADIK	80	0.43	-0.20	0.35
GRP78	ELEEIVQPIISK	59	0.52	-0.20	0.43
C1s	TNFDNDIALVR	80	0.55	-0.29	0.26
GRP78	NELESYAYSLK	52	0.86	-0.54	0.14
GAPDH	VGVNGFGR	80	0.88	-0.48	0.050
V-ATPase	ASLYPCPETPQER	69	0.89	-0.22	0.35
V-ATPase	EINTNQEALK	70	0.95	-0.28	0.074
GAPDH	GALQNIIPASTGAAK	80	0.96	-0.46	0.013
GAPDH	AGAHLQGGAK	75	0.96	-0.50	0.0045
V-ATPase	DLNPDVNVFQR	80	0.98	-0.27	0.15
IGHV	TGYYWGWIR	0			

Note: The trend of each peptide with the stage of liver fibrosis was assessed by the Jonckheer-Terpstra test. The difference between patients with F1-2 and those with F3-4 for each peptide was assessed by the Mann–Whitney U test.

Abbreviations: SAP, serum amyloid P component; PPBP, pro-platelet basic protein; Lp (a), lipoprotein (a); V-ATPase, ATPase H+ transporting V0 subunit a1; GRP78, 78 kDa glucose-regulated protein; IGHV, Ig heavy chain V-II region WAH; C1s, complement C1s subcomponent; GAPDH, glyceraldehyde-3-phosphate dehydrogenase; CD9, CD9 antigen

### Diagnostic accuracies of EV protein levels for severe fibrosis

We compared the diagnostic accuracies for severe fibrosis between the EV protein levels assessed by the target proteomic analysis and other liver fibrosis markers, namely, type IV collagen 7S, hyaluronic acid, and the FIB-4 index, using the validation cohort. The AUCs of all the SAP-targeted peptides and one PPBP-targeted peptide to diagnose patients with F≥3 were approximately 0.7–0.8 (S2 Fig). The AUCs of SAP-targeted peptides to diagnose patients with F≥3 were comparable to those of type IV collagen 7S, hyaluronic acid, and the FIB-4 index (Table 3). The AUC of the PPBP-targeted peptide to diagnose patients with F≥3 was also comparable to the AUCs of hyaluronic acid and the FIB-4 index (Table 3).

**Table 3:** Results of the statistical comparisons for AUCs of the verified peptides with those of other markers.

Factor	AUC	P value vs. AYSLFSYNTQGR	P value vs. DNELLVYK	P value vs. IVLGQEQDSYGGK	P value vs. ICLDPDAPR
AYSLFSYNTQGR (SAP)	0.769	reference	0.62	0.52	0.054
DNELLVYK (SAP)	0.772	0.62	reference	0.76	0.039
IVLGQEQDSYGGK (SAP)	0.774	0.52	0.76	reference	0.034
ICLDPDAPR (PPBP)	0.688	0.054	0.039	0.034	reference
Type IV collagen 7S	0.823	0.26	0.28	0.30	0.0099
Hyaluronic acid	0.764	0.93	0.87	0.85	0.24
FIB-4 index	0.739	0.48	0.42	0.41	0.31

Note: The AUC of each peptide was used as a reference and compared with the AUCs of the other peptides, type IV collagen 7S, hyaluronic acid, and the FIB-4 index by the Delong test.

Abbreviations: AUC, area under the curve; SAP, serum amyloid P component; PPBP, pro-platelet basic protein; FIB-4 index, fibrosis-4 index

### Serum SAP and PPBP levels correlate with EV SAP and PPBP levels and decrease with the progression of liver fibrosis in patients with chronic hepatitis C

**Fig 3:**
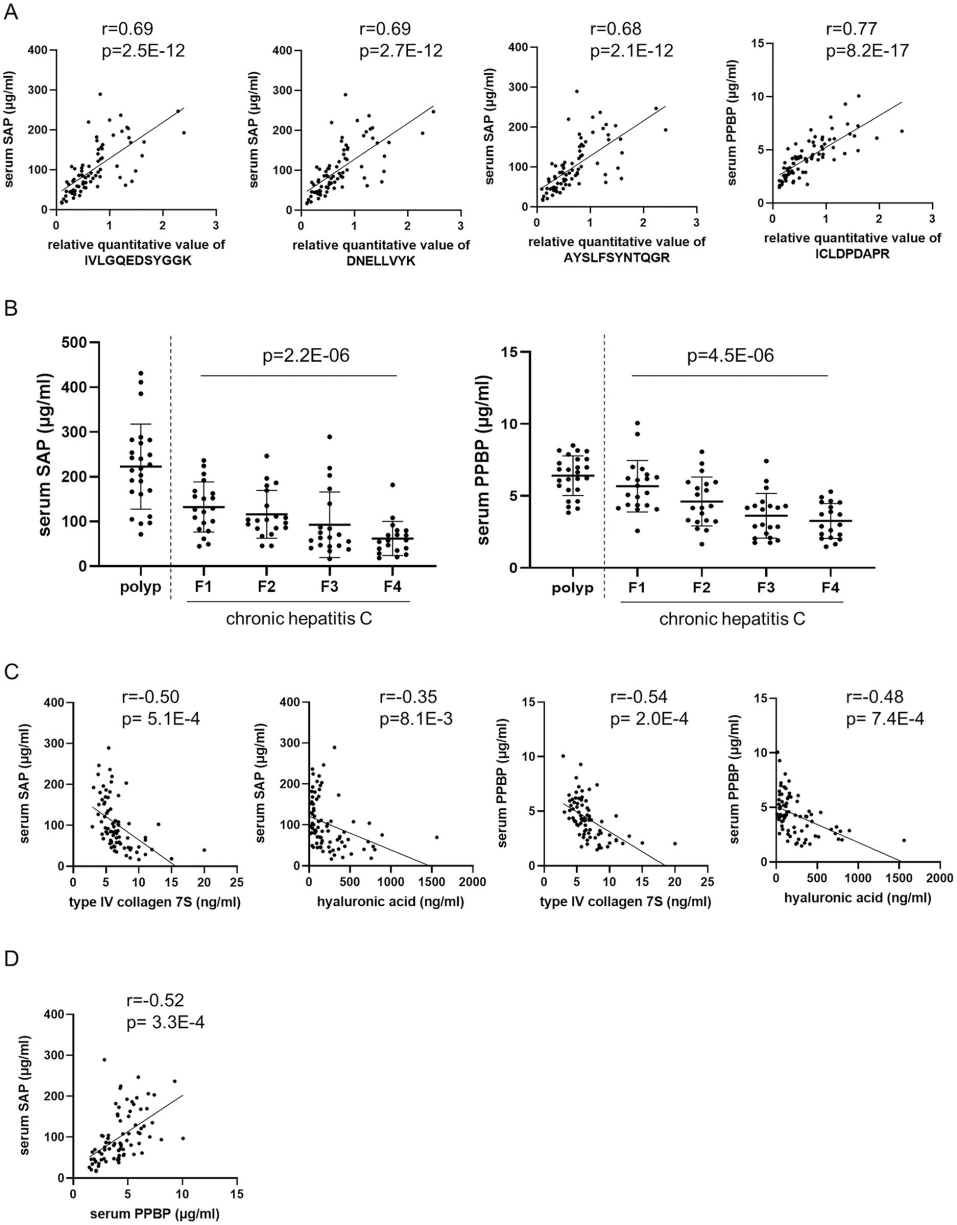
The results of serum SAP and PPBP measurements in patients with chronic hepatitis C. (A) The correlations between serum SAP or PPBP and their corresponding peptides evaluated by the Pearson product-moment correlation coefficient. (B) Serum SAP and PPBP levels in patients with chronic hepatitis C from the validation cohort. Levels in patients with colorectal polyps without liver disease are shown as controls. The dot plot shows individual values. The data are expressed as the mean ± standard deviation. The p-values calculated by the Jonckheere-Terpstra test for the trend in patients with chronic hepatitis C are shown. (C) The correlations of serum SAP or PPBP levels with type IV collagen 7S and hyaluronic acid levels evaluated by calculating the Pearson product-moment correlation coefficient. (D) The correlations between serum SAP and PPBP levels evaluated by calculating the Pearson product-moment correlation coefficient. Abbreviations: SAP, serum amyloid P component; PPBP, pro-platelet basic protein.

Both SAP and PPBP are secreted proteins [25, 26]. As fibrosis markers, it is more useful if they can be evaluated in serum rather than in EVs. Therefore, we examined whether serum SAP and PPBP could be detected by ELISA kits. Serum SAP and PPBP levels in patients with chronic hepatitis C before DAA treatment could be measured by ELISAs and were highly positively correlated with the EV SAP and PPBP levels measured by targeted proteomics (Fig 3A). Their expression levels tended to decrease significantly with the progression of liver fibrosis (Fig 3B). We compared the serum SAP and PPBP levels before DAA treatment with those obtained at 24 weeks or 72 weeks after the end of treatment, when the hepatitis C virus was eliminated. Serum SAP and PPBP levels did not differ between before DAA treatment and 24 or 72 weeks after the end of treatment (S3 Fig).

Next, we also measured serum SAP and PPBP levels in patients with colorectal polyps without liver disease as a control group (Fig 3B) and in patients with chronic hepatitis B and nonalcoholic fatty liver disease (NAFLD) as a group of patients with other liver diseases. In patients with colorectal polyps without liver disease, serum SAP and PPBP levels were both significantly higher than those in patients with chronic hepatitis C (p = 7.1E-10, 8.7E-7, respectively. Mann–Whitney U tests) (Fig 3B). Serum SAP levels also significantly decreased with the progression of liver fibrosis in both patients with chronic hepatitis B and patients with NAFLD (S4A and S4B Fig). On the other hand, serum PPBP levels were significantly decreased with the progression of liver fibrosis in patients with NAFLD, but not in patients with chronic hepatitis B (S4A and S4B Fig).

### Diagnostic accuracies of serum SAP and PPBP for liver fibrosis stage

**Fig 4:**
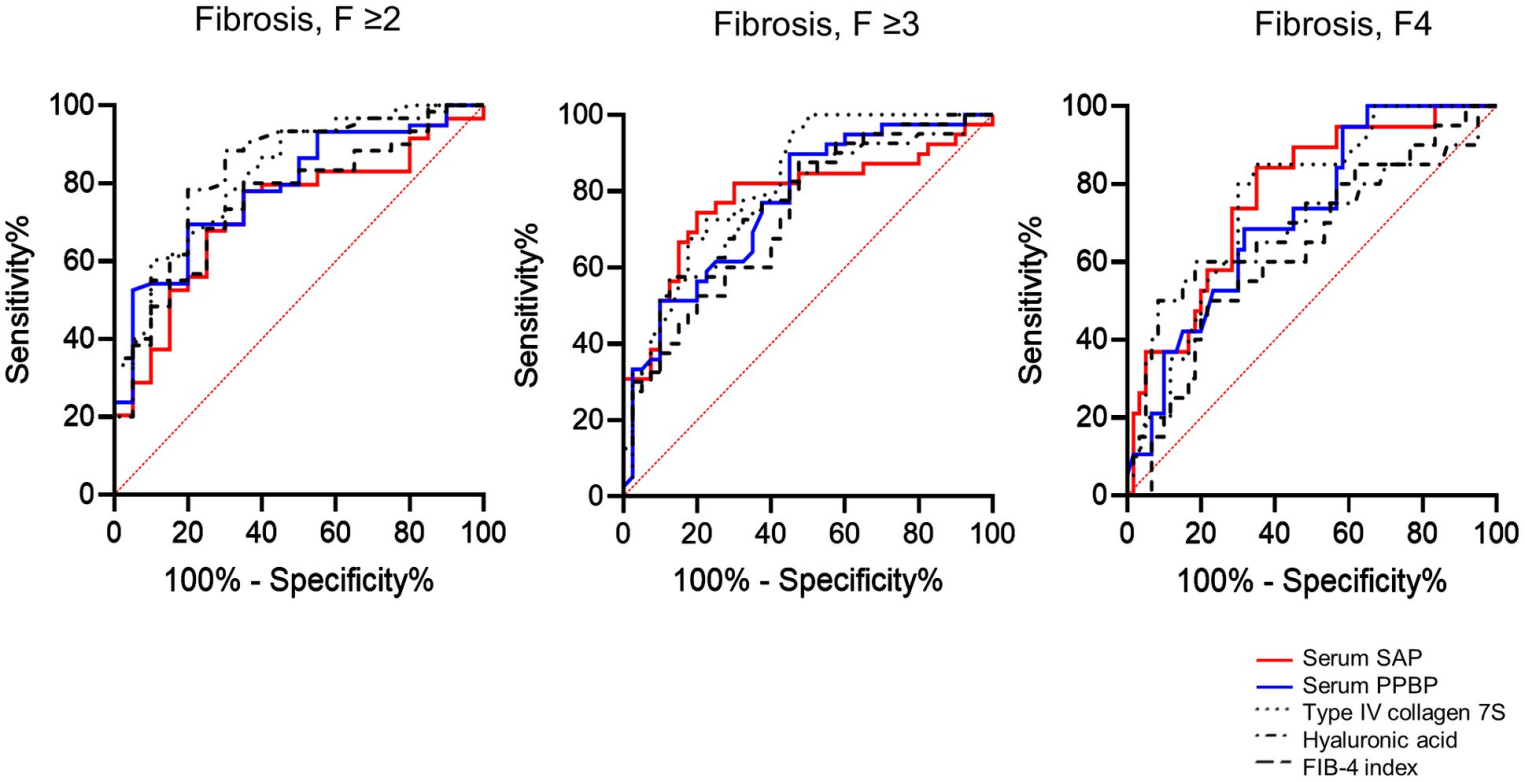
Comparison of diagnostic accuracies for liver fibrosis stage. The receiver operating characteristic curves of serum SAP, serum PPBP, type IV collagen 7S, hyaluronic acid, and the FIB-4 index for diagnosing patients with F≥2, those with F≥3 and those with F4 are shown. Abbreviations: SAP, serum amyloid P component; PPBP, pro-platelet basic protein; FIB-4 index, fibrosis-4 index.

We evaluated the correlations among serum SAP and PPBP levels and the levels of other reported liver fibrosis markers, namely, type IV collagen 7S and hyaluronic acid. Both serum SAP levels and serum PPBP levels showed some degree of negative correlation with serum type IV collagen 7S levels and a weak negative correlation with serum hyaluronic acid levels (Fig 3C). Serum SAP levels exhibited some degree of positive correlation with serum PPBP levels (Fig 3D). Similar to SAP and PPBP in EVs, we also compared the ability of SAP and PPBP in serum to diagnose fibrosis stage with the abilities of other fibrosis markers. The AUCs of serum SAP and PPBP for diagnosing patients with F≥3 were 0.78 and 0.769, respectively, and were comparable to those of type IV collagen 7S, hyaluronic acid, and the FIB-4 index (Fig 4, Table 4). Moreover, the AUCs of serum SAP and PPBP for diagnosing patients with F≥2 and F4 were also comparable to those of type IV collagen 7S, hyaluronic acid, and the FIB-4 index (Table 4). Although the AUCs did not significantly increase for combination of SAP with PPBP compared to SAP or PPBP alone (data not shown), both SAP and PPBP had significantly higher AUCs than the FIB-4 index, especially in diagnosing patients with stage F4 fibrosis.

**Table 4:** Results of statistical comparisons of AUCs for diagnosing each liver fibrosis stage.

Factor	F≥2, AUC	F≥2 P value vs. serum SAP	F≥2 P value vs. serum PPBP	F≥3, AUC	F≥3 P value vs. serum SAP	F≥3 P value vs. serum PPBP	F4, AUC	F4 P value vs. serum SAP	F4 P value vs. serum PPBP
Serum SAP	0.722	reference	0.28	0.780	reference	0.85	0.770	reference	0.36
Serum PPBP	0.786	0.28	reference	0.769	0.85	reference	0.719	0.36	reference
Type IV collagen 7S	0.826	0.051	0.54	0.823	0.48	0.32	0.756	0.68	0.63
Hyaluronic acid	0.833	0.12	0.54	0.764	0.77	0.88	0.692	0.23	0.64
FIB-4 index	0.762	0.53	0.64	0.739	0.42	0.39	0.635	0.011	0.038

Note: The AUC of serum SAP or serum PPBP was used as a reference and compared with the AUCs of type IV collagen 7S, hyaluronic acid, and the FIB-4 index by the Delong test.

Abbreviations: AUC, area under the curve; SAP, serum amyloid P component; PPBP, pro-platelet basic protein; FIB-4 index, fibrosis-4 index

## Discussion

In the present study, we identified SAP and PPBP in EVs as candidate biomarkers for diagnosing liver fibrosis in chronic hepatitis C. In addition, we clarified that EV SAP and PPBP levels are strongly correlated with serum SAP and PPBP levels and that serum SAP and PPBP could also be markers of liver fibrosis.

EVs are nanosized vesicles that are secreted from various types of human cells and contain proteins, mRNAs and microRNAs [12]. EVs contain not only a conserved set of common proteins essential for vesicle biogenesis, structure, and transport but also proteins that change in response to physiological and pathological conditions [13]. The proteins in EVs are derived from the cytoplasm, nucleus, cell membrane and cytoskeleton and may differ in composition from proteins in serum [27]. For example, type IV collagen 7S, which is present in the basement membrane and clinically used as a fibrosis marker, has not been reported in the profile of serum-derived EVs thus far [28, 29], including in our present study. In addition, EV isolation allows the detection of proteins that otherwise would be impossible to detect in serum given the presence of very abundant proteins such albumin [30]. Therefore, EVs are an excellent source for biomarkers. Several comprehensive analyses of EV proteins have been performed to detect liver cirrhosis biomarkers [31–33], but these studies have not evaluated EV protein changes due to stepwise fibrosis progression. We performed a comprehensive analysis of serum-derived EV proteins in chronic hepatitis C patients and clarified for the first time that SAP and PPBP in EVs could be markers of liver fibrosis.

In the present study, we found that serum SAP levels were decreased with the progression of liver fibrosis (Fig 3). SAP is a member of the pentraxin family that includes C-reactive protein and pentraxin 3. SAP is synthesized by the liver and circulates in the blood as a stable pentamer [25]. SAP is implicated in the activation of the classical complement pathway and the clearance of DNA, chromatin and apoptotic cells [34]. SAP is codeposited with amyloid fibrils in all types of amyloidosis, including cerebral beta-protein amyloid deposits associated with Alzheimer’s disease [35]. Low SAP levels in cerebrospinal fluid have been reported to represent a risk of progression to Alzheimer’s disease in patients with mild cognitive impairment [36]. Serum SAP levels in patients with major depressive disorder [37], amphetamine addicts [38], Parkinson’s disease [38] and proliferative diabetic retinopathy [39] are reported to be higher than those in healthy controls. On the other hand, serum SAP levels in patients with idiopathic pulmonary fibrosis [40] and paraquat poisoning [41] are reported to be lower than those in healthy controls. For liver disease, patients with acute hepatitis and liver cirrhosis due to hepatitis B virus showed lower serum SAP levels than healthy controls [42]. Patients with NAFLD showed lower serum SAP levels than healthy controls, and those with advanced fibrosis showed much lower serum SAP levels [43]. Consistent with these reports, serum SAP levels decreased significantly with the progression of liver fibrosis in both patients with chronic hepatitis B and patients with NAFLD (S4A and S4B Fig). However, there is no previous report on hepatitis C, and we first revealed that serum SAP levels decrease with the progression of liver fibrosis in hepatitis C. SAP inhibits the differentiation of monocyte-derived fibroblast-like cells called fibrocytes and neutrophil adhesion to extracellular matrix proteins [44]. Although the detailed mechanism by which SAP is involved in liver fibrosis remains unclear, SAP suppresses leukocyte infiltration and hepatic stellate cell activation in a mouse model of acute liver injury induced by carbon tetrachloride [45]. Based on this result, reduced levels of SAP, which inhibits hepatic stellate cell activation, might contribute to the development of liver fibrosis.

PPBP belongs to the CXC chemokine family and has been reported to be involved in various cellular processes, including DNA synthesis, mitosis, glycolysis and intracellular cyclic adenosine monophosphate accumulation [46]. Previous studies have shown increased serum levels of PPBP in cardiovascular disease, stroke and lung cancer [47–49]. However, serum PPBP levels have been reported to be decreased in pancreatic cancer and ovarian cancer [50, 51]. In clear cell renal carcinoma, serum PPBP has been reported to be a predictive marker of sunitinib efficacy [52]. For organ fibrosis, serum PPBP levels are reported to be increased in patients with idiopathic pulmonary fibrosis compared to healthy controls [53], but there are still no reports on liver fibrosis. PPBP is a precursor to a wide variety of derivatives, including thrombocidin, neutrophil-activating peptide-2, and connective tissue–activating peptide-III [54]. Connective tissue–activating peptide-III stimulates the synthesis of DNA, hyaluronic acid, glycosaminoglycans, and proteoglycan core protein in human fibroblasts [55], implying that PPBP might facilitate fibrogenesis. However, in the present study, PPBP levels decreased with liver fibrosis progression, and we postulate that PPBP does not exert a substantial effect on fibrosis formation, but simply reflects fibrosis. PPBP is a small protein produced by monocytes, macrophages, and mainly platelets [56], and thus the decrease in PPBP levels in EVs and serum may be associated with the decrease in platelet counts due to the progression of liver fibrosis. In addition, PPBP expression is reportedly regulated by the anti-inflammatory cytokine IL-10 [56], suggesting that the decrease in PPBP levels with the progression of liver fibrosis also reflects the increase in IL-10 production in response to the progression of fibrosis. Although the relationship between PPBP levels and platelet counts or IL-10 levels requires further investigation, we have revealed for the first time that serum PPBP levels decrease with the progression of liver fibrosis.

In the present study, serum SAP and PPBP levels did not differ between pretreatment and 24 or 72 weeks after the end of treatment (S3 Fig). On the other hand, serum type IV collagen 7S and hyaluronic acid levels decreased significantly from pretreatment to 24 or 72 weeks after the end of treatment (S3 Fig). Existing direct serum biomarkers of fibrosis, including type IV collagen 7S and hyaluronic acid, are reported to tend to be elevated to a greater extent when associated with high inflammatory activity [57]. In contrast to these existing direct serum biomarkers of fibrosis, serum SAP and PPBP levels may not be affected by inflammatory activity in the liver.

Several serum proteomic analyses have identified novel fibrosis markers in chronic hepatitis C patients. In those studies, proteome analysis based on the two-dimensional gel electrophoresis method and the isobaric tags for relative and absolute quantitation labeling method was performed [58–61]. Two-dimensional gel electrophoresis-based proteomics studies identified apolipoprotein L1 and J, mac2 binding protein, and vitamin D-binding protein as novel fibrosis markers [58–60], whereas SAP and PPBP were not identified in those studies. PPBP has been identified as a marker candidate protein in isobaric tags for relative and absolute quantitation labeling-based proteomics studies but has not been validated [61]. In the present study, we identified serum SAP and PPBP as fibrosis markers for the first time by performing proteomic analysis of EV proteins. For secreted proteins, there is one report showing that there was a correlation between CD44 levels in EVs and those in serum [62]. It has been suggested that targeting proteins in EVs would not only identify new markers in EVs but may also allow candidate proteins to be applied as markers in serum, especially if the identified protein is a secreted protein.

## Conclusions

We identified that SAP and PPBP levels in EVs decreased as liver fibrosis progressed by proteomic analysis in patients with chronic hepatitis C. Serum SAP and PPBP levels were strongly correlated with those in EVs and decreased as liver fibrosis progressed. SAP and PPBP levels in serum as well as in EVs are novel biomarkers of liver fibrosis in chronic hepatitis C patients.

## Supporting information

S1 Fig Study flowchart for enrollment in the trial cohort and the validation cohort. Abbreviations: HCC, hepatocellular carcinoma; HBV, hepatitis B virus; SVR, sustained virologic response.
(TIF)

S2 Fig The receiver operating characteristic curve analysis of IVLGQEDSYGGK, DNELLVYK, AYSLFSYNTQGR, and ICLDPDAPR. The receiver operating characteristic curves of IVLGQEDSYGGK, DNELLVYK, AYSLFSYNTQGR, and ICLDPDAPR are shown. Abbreviations: AUC, area under the curve.
(TIF)

S3 Fig Changes after HCV elimination. In-time measurements of serum SAP, PPBP, type IV collagen 7S and hyaluronic acid levels over time in 47 patients with chronic hepatitis C from the validation cohort whose sera were available at both 24 weeks and 72 weeks after the end of treatment. Each line represents individual values. The p-values were calculated using the Wilcoxon signed-rank sum test between those before DAA treatment and those at 24 or 72 weeks after the end of treatment. *: p<0.01.
(TIF)

S4 Fig Serum SAP and PPBP levels in patients with chronic hepatitis B and patients with NAFLD. Serum SAP and PPBP levels in patients with chronic hepatitis B (n = 97) (A) and patients with NAFLD (n = 62) (B). The dot plot shows individual values. The data are presented as the mean ± standard deviation. The p-values calculated using the Jonckheere-Terpstra test are shown. Abbreviations: PPBP, pro-platelet basic protein; SAP, serum amyloid P component.
(TIF)

S1 Table The characteristics of patients in the trial cohort. (DOCX)

S2 Table The characteristics of patients in the validation cohort. (DOCX)

S1 File Supplementary materials. (DOCX)

## Abbreviations

AUC: area under the curve
C1s: complement C1s subcomponent
CD9: CD9 antigen
DAA: direct-acting antiviral
ELISA: enzyme-linked immunosorbent assay
EV: extracellular vesicle
FIB-4 index: fibrosis-4 index
GAPDH: glyceraldehyde-3-phosphate dehydrogenase
GRP78: 78 kDa glucose-regulated protein
IGHV: Ig heavy chain V-II region WAH
Lp (a): lipoprotein (a)
NAFLD: nonalcoholic fatty liver disease
PPBP: pro-platelet basic protein
SAP: serum amyloid P component
SRM: selected reaction monitoring
TMT: tandem mass tag
V-ATPase: ATPase H+ transporting V0 subunit a1
