# Supplementary figures and images for: Serum amyloid P component and pro-platelet basic protein in extracellular vesicles or serum are novel markers of liver fibrosis in chronic hepatitis C patients
Source: PLoS One. 2022 Jul 7;17(7):e0271020. doi: 10.1371/journal.pone.0271020 (PMC9262231; doi:10.1371/journal.pone.0271020)

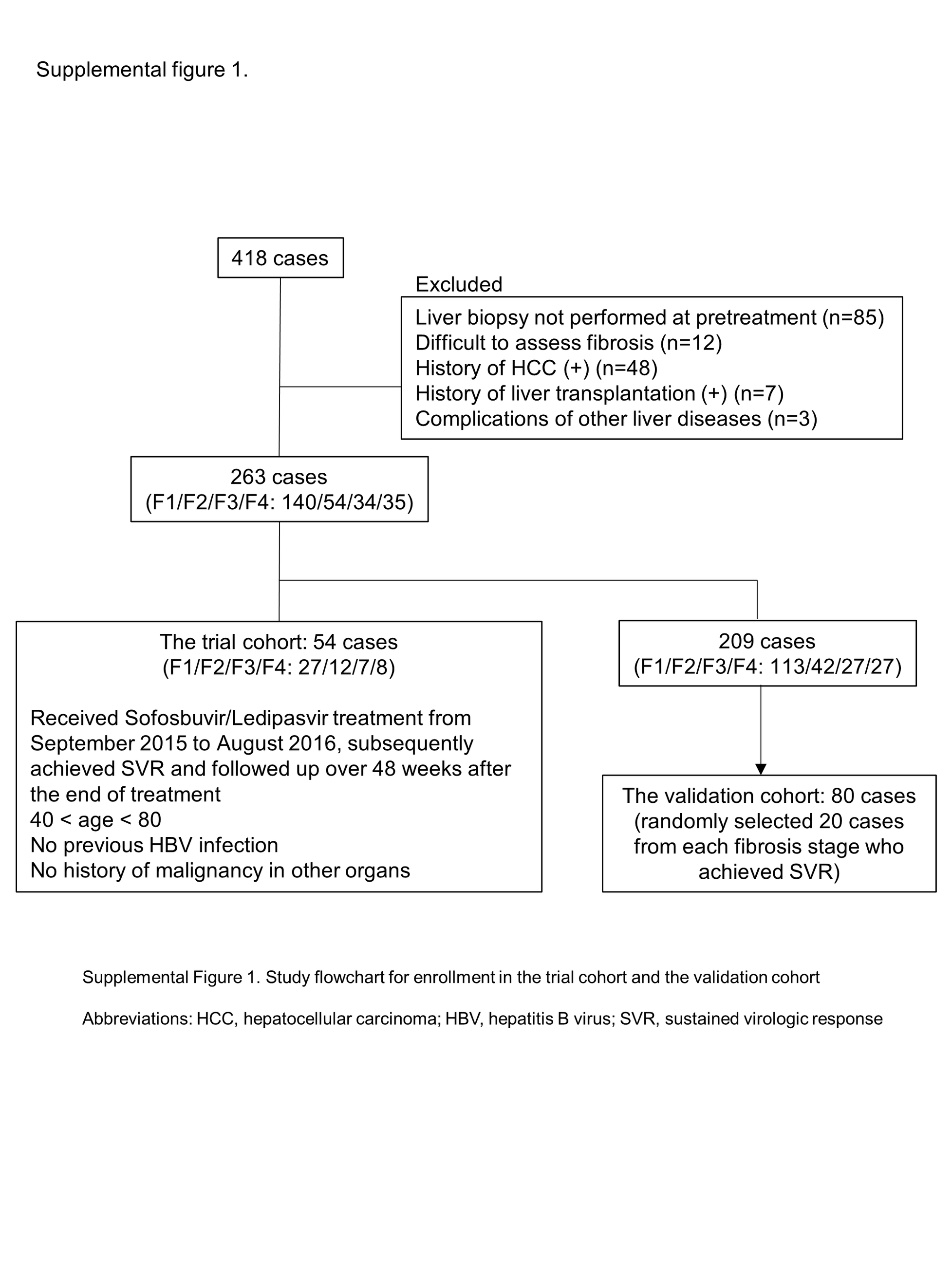

Supplement: S1 Fig — Abbreviations: HCC, hepatocellular carcinoma; HBV, hepatitis B virus; SVR, sustained virologic response. (TIF) [file pone.0271020.s001.tif]

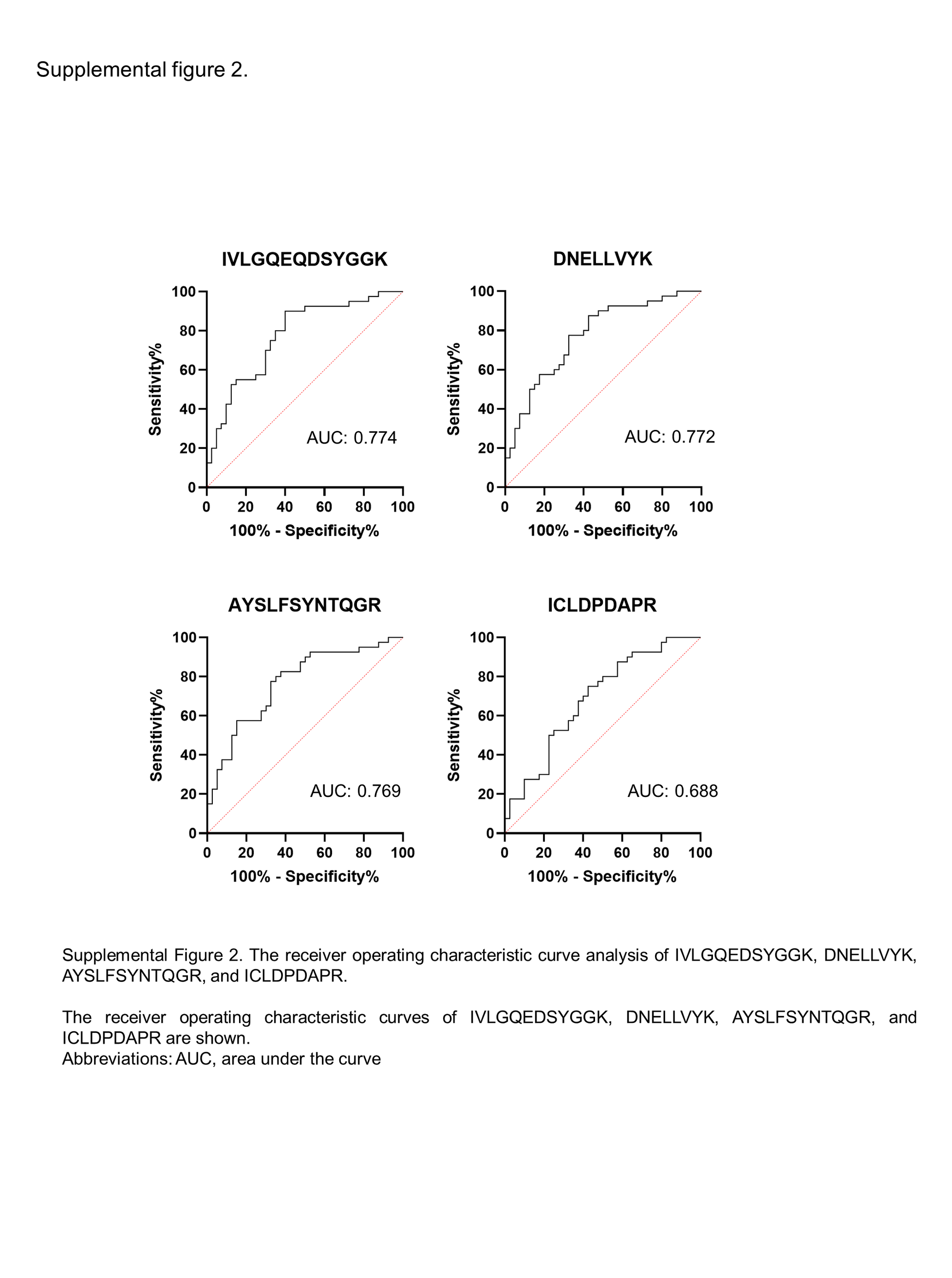

Supplement: S2 Fig — The receiver operating characteristic curves of IVLGQEDSYGGK, DNELLVYK, AYSLFSYNTQGR, and ICLDPDAPR are shown. Abbreviations: AUC, area under the curve. (TIF) [file pone.0271020.s002.tif]

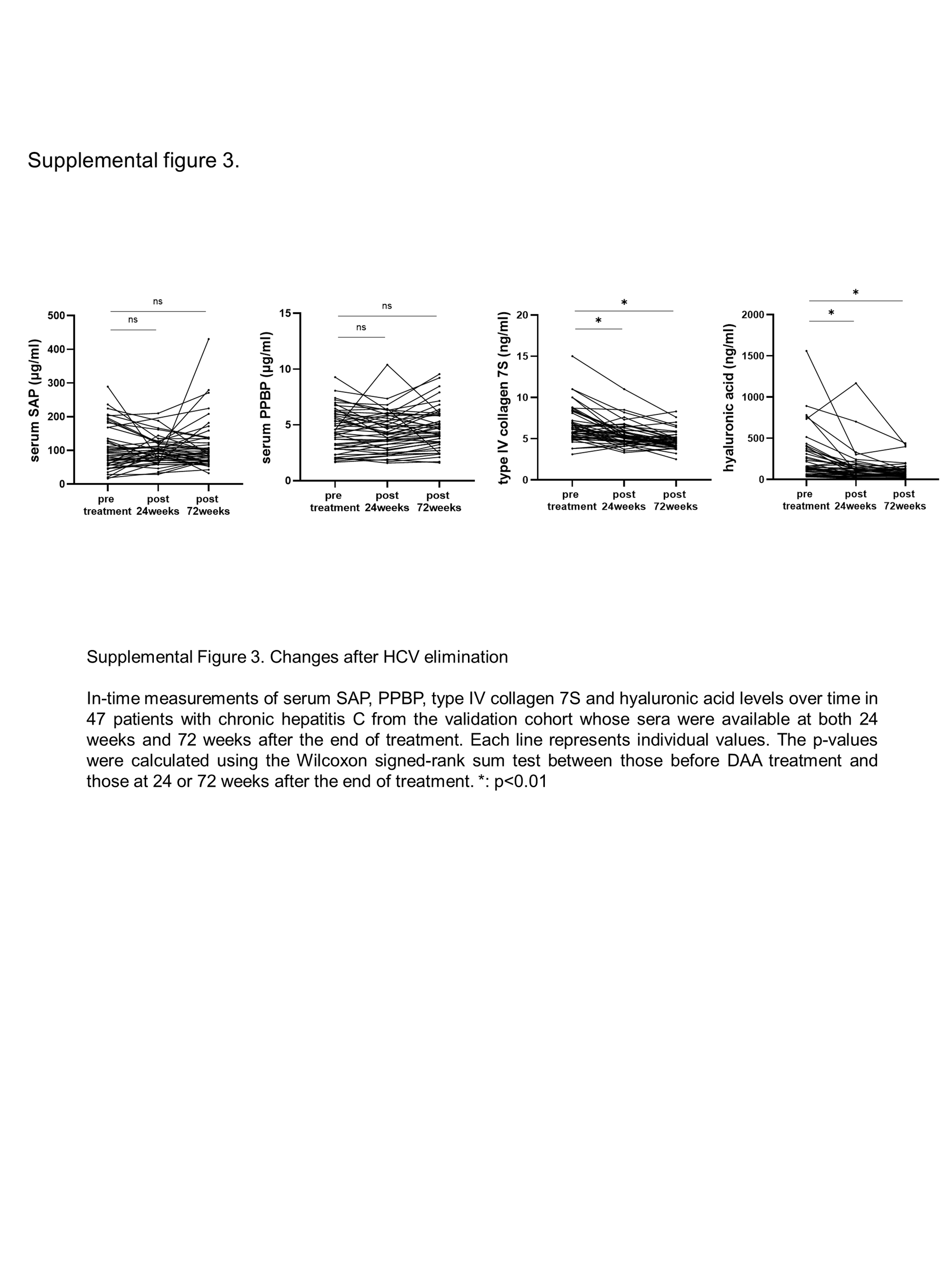

Supplement: S3 Fig — In-time measurements of serum SAP, PPBP, type IV collagen 7S and hyaluronic acid levels over time in 47 patients with chronic hepatitis C from the validation cohort whose sera were available at both 24 weeks and 72 weeks after the end of treatment. Each line represents individual values. The p-values were calculated using the Wilcoxon signed-rank sum test between those before DAA treatment and those at 24 or 72 weeks after the end of treatment. *: p<0.01. (TIF) [file pone.0271020.s003.tif]

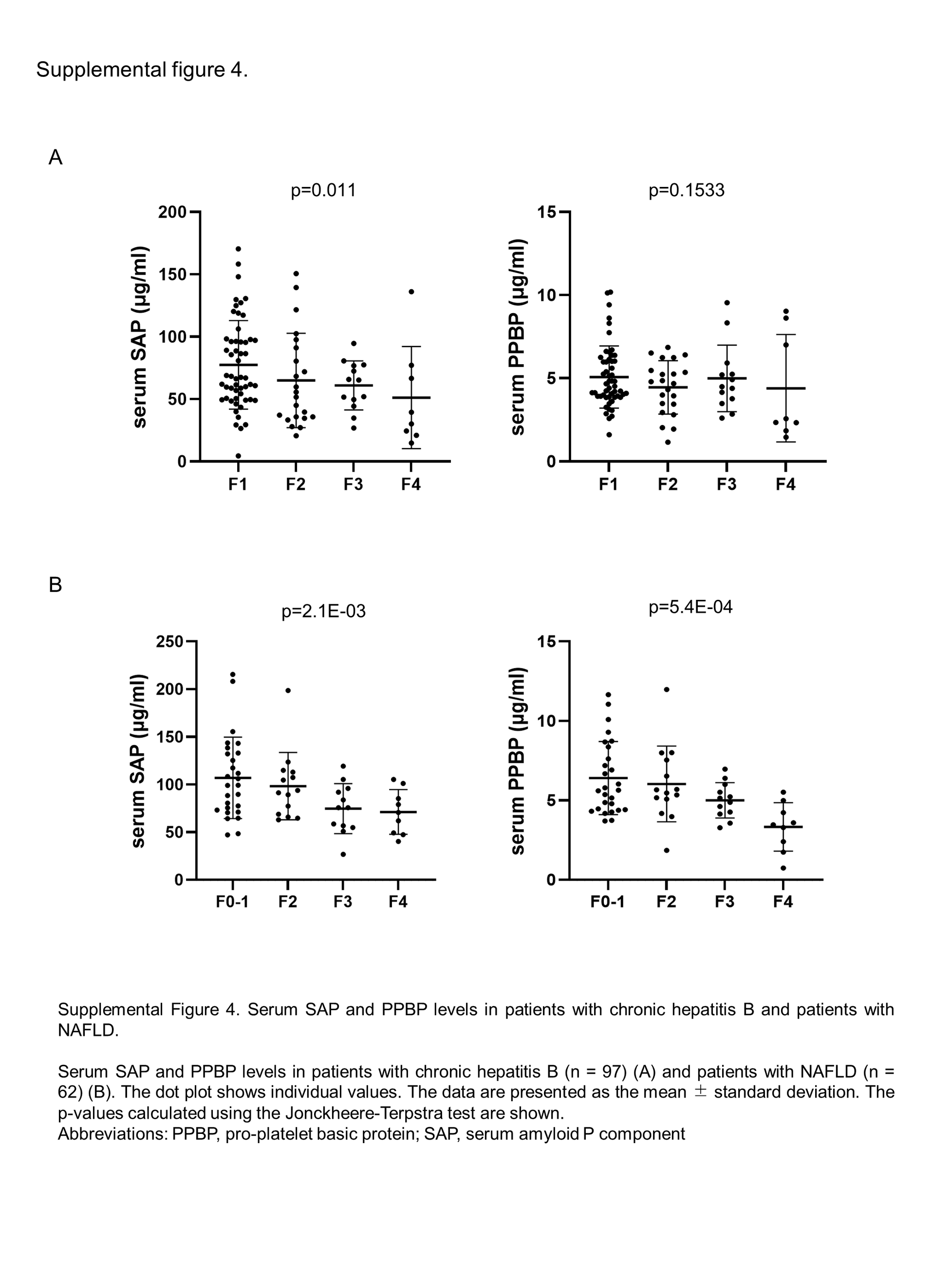

Supplement: S4 Fig — Serum SAP and PPBP levels in patients with chronic hepatitis B (n = 97) (A) and patients with NAFLD (n = 62) (B). The dot plot shows individual values. The data are presented as the mean ± standard deviation. The p-values calculated using the Jonckheere-Terpstra test are shown. Abbreviations: PPBP, pro-platelet basic protein; SAP, serum amyloid P component. (TIF) [file pone.0271020.s004.tif]
