# Supplementary material for: Serum amyloid P component and pro-platelet basic protein in extracellular vesicles or serum are novel markers of liver fibrosis in chronic hepatitis C patients
Source: PLoS One. 2022 Jul 7;17(7):e0271020. doi: 10.1371/journal.pone.0271020 (PMC9262231; doi:10.1371/journal.pone.0271020)
Supplement: S1 Table — (DOCX) [file pone.0271020.s005.docx]

|  | F1 | F2 | F3 | F4 | P value |
| --- | --- | --- | --- | --- | --- |
| Number | 27 | 12 | 7 | 8 |  |
| Age (years) | 67 (58-72) | 68 (56-74) | 66 (51-67) | 68 (61-72) | 0.52 |
| Sex (male/female) | 10/17 | 6/6 | 3/4 | 4/4 | 0.82 |
| BMI (kg/m^2^) | 22.4 (20.3-23.5) | 20.4 (19.8-22.3) | 21.8 (20.7-22.5) | 24.0 (20.8-27) | 0.21 |
| HCV-RNA (Log IU/ml) | 6.0 (5.5-6.5) | 6.1 (5.7-6.3) | 6.0 (5.5-6.5) | 6.4 (6.3-6.5) | 0.46 |
| Platelets (×10^4^/μl) | 22.7 (17.9-24.8) | 14.7 (13.4-17.8) | 12.0 (11.1-13.8) | 11.4 (9.5-12.7) | <0.001 |
| Total bilirubin (mg/dl) | 0.6 (0.5-0.7) | 0.7 (0.6-1.0) | 0.5 (0.5-0.7) | 1.0 (1.0-1.2) | 0.0083 |
| Alb (g/dl) | 4.0 (3.9-4.3) | 4.1 (3.7-4.3) | 3.8 (3.6-4.0) | 3.6 (3.4-4.1) | 0.12 |
| PT (%) | 91 (88-98) | 86 (85-90) | 86 (81-93) | 85 (76-88) | 0.033 |
| AST (U/l) | 34 (29-39) | 39 (34-57) | 44 (32-53) | 82 (62-97) | 0.0018 |
| ALT (U/l) | 34 (20-49) | 39 (33-51) | 49 (27-62) | 59 (49-121) | 0.019 |
| γ-GTP (U/l) | 22 (17-42) | 30 (22-45) | 43 (22-53) | 42 (35-82) | 0.062 |
| AFP (ng/ml) | 3 (3-4) | 4 (3-8) | 5 (3-11) | 24 (9-58) | 0.0023 |
| PIVKA-II (mAU/ml) | 21 (16-25) | 26 (18-30) | 23 (20-24) | 22 (17-26) | 0.62 |
| FIB-4 index | 1.90 (1.50-2.90) | 2.42 (2.11-4.84) | 2.23 (2.01-4.73) | 6.53 (3.85-8.51) | <0.001 |
| Hyaluronic acid (ng/ml) | 77 (44-100) | 80 (46-137) | 157 (90-192) | 240 (117-468) | 0.024 |
| Type IV collagen 7S (ng/ml) | 4.8 (4.3-5.7) | 5.5 (4.6-7.0) | 5.6 (5.1-7.2) | 11.0 (8.2-11.5) | 0.0026 |

Supplemental Table1. The characteristics of patients in the trial cohort

Note: All quantitative data are presented as medians (interquartile range), unless stated otherwise.

Abbreviations: BMI, body mass index; Alb, albumin; PT, prothrombin time; AST, aspartate aminotransferase; ALT, alanine aminotransferase; γ-GTP, γ-glutamyl transpeptidase; AFP, alpha-fetoprotein; PIVKA-II, protein induced by vitamin K absence or antagonist II; FIB-4 index, fibrosis-4 index
