# Supplementary material for: Serum amyloid P component and pro-platelet basic protein in extracellular vesicles or serum are novel markers of liver fibrosis in chronic hepatitis C patients
Source: PLoS One. 2022 Jul 7;17(7):e0271020. doi: 10.1371/journal.pone.0271020 (PMC9262231; doi:10.1371/journal.pone.0271020)
Supplement: S2 Table — (DOCX) [file pone.0271020.s006.docx]

|  |  | F1 | F2 | F3 | F4 | P value |
| --- | --- | --- | --- | --- | --- | --- |
| Number |  | 20 | 20 | 20 | 20 |  |
| Age (years) |  | 68 (59-71) | 66 (58-74) | 68 (62-74) | 73 (64-76) | 0.40 |
| Sex (male/female) |  | 9/11 | 7/13 | 9/11 | 6/14 | 0.70 |
| BMI (kg/m^2^) |  | 23.2 (22.0-24.9) | 22.5 (21.0-25.7) | 20.7 (20.1-23.9) | 23.7 (20.5-24.2) | 0.50 |
| HCV-RNA (Log IU/ml) |  | 6.5 (5.9-6.7) | 6.1 (5.3-6.4) | 6.3 (5.7-6.6) | 6.3 (6.1-6.6) | 0.13 |
| Treatment method | DCV/ASV | 0 | 1 | 3 | 2 | 0.62 |
|  | SOF/LDV | 11 | 15 | 11 | 11 |  |
|  | SOF/RBV | 5 | 3 | 1 | 3 |  |
|  | OBV/PTV/r | 0 | 1 | 1 | 1 |  |
|  | EBR/GZR | 1 | 0 | 2 | 1 |  |
|  | GLE/PIB | 3 | 0 | 2 | 2 |  |
| Platelets (×10^4^/μl) |  | 17.8 (16.0-21.9) | 17.0 (11.4-22.9) | 14.4 (8.2-16.7) | 11.5 (8.3-16.3) | 0.0017 |
| Total bilirubin (mg/dl) |  | 0.7 (0.6-0.8) | 0.7 (0.5-1.0) | 0.7 (0.6-0.9) | 0.7 (0.6-0.9) | 0.89 |
| Alb (g/dl) |  | 4.1 (3.8-4.3) | 4.0 (3.8-4.2) | 3.7 (3.5-4.1) | 3.9 (3.5-4.1) | 0.036 |
| PT (%) |  | 91 (85-96) | 87 (81-95) | 80 (75-84) | 82 (74-86) | 0.0022 |
| AST (U/l) |  | 30 (25-47) | 52 (38-82) | 57 (46-93) | 47 (30-52) | <0.001 |
| ALT (U/l) |  | 26 (17-48) | 57 (38-93) | 47 (38-93) | 36 (28-60) | 0.0066 |
| γ-GTP (U/l) |  | 21 (16-37) | 40 (30-54) | 42 (29-60) | 24 (20-37) | 0.0024 |
| AFP (ng/ml) |  | 4 (3-6) | 6 (5-7) | 11 (5-22) | 10 (4-13) | 0.0022 |
| PIVKA-II (mAU/ml) |  | 19 (15-26) | 24 (17-30) | 20 (17-28) | 19 (16-26) | 0.36 |
| FIB-4 index |  | 2.07 (1.75-2.67) | 3.25 (1.83-4.41) | 3.78 (2.58-8.46) | 4.27 (2.43-6.35) | <0.001 |
| Hyaluronic acid (ng/ml) |  | 49 (38-95) | 132 (79-228) | 255 (135-350) | 378 (120-575) | <0.001 |
| Type IV collagen 7S(ng/ml) |  | 4.8 (4.2-5.8) | 5.8 (4.9-6.5) | 6.9 (5.8-9.0) | 7.1 (6.6-8.7) | <0.001 |

Supplemental Table2. The characteristics of patients in the validation cohort

Note: All quantitative data are presented as medians (interquartile range), unless stated otherwise.

Abbreviations: BMI, body mass index; DCV, daclatasvir; ASV, asunaprevir; SOF, sofosbuvir; LDV, ledipasvir; RBV, ribavirin; OBV, ombitasvir; PTV, paritaprevir; r, ritonavir; EBR, elbasvir; GZR, grazoprevir; GLE, glecaprevir; PIB, pibrentasvir; Alb, albumin; PT, prothrombin time; AST, aspartate aminotransferase; ALT, alanine aminotransferase; γ-GTP, γ-glutamyl transpeptidase; AFP, alpha-fetoprotein; PIVKA-II, protein induced by vitamin K absence or antagonist II; FIB-4 index, fibrosis-4 index
