# Supplementary material for: Serum amyloid P component and pro-platelet basic protein in extracellular vesicles or serum are novel markers of liver fibrosis in chronic hepatitis C patients
Source: PLoS One. 2022 Jul 7;17(7):e0271020. doi: 10.1371/journal.pone.0271020 (PMC9262231; doi:10.1371/journal.pone.0271020)
Supplement: S1 File — (DOCX) [file pone.0271020.s007.docx]

**Supplementary materials**

**The protocol of nontargeted proteomics**

The Tandem mass Tag labeled peptides were analyzed by a Q-Exactive plus mass spectrometer (Thermo Scientific, Bremen, Germany) with an UltiMate 3000 Nanoflow high-performance LC system (Dionex, Sunnyvale, CA) and an HTC-PAL autosampler (CTC Analytics, Zwingen, Switzerland). The analytical column was packed with the reversed-phase material ReproSil-Pur C18-AQ, 1.9-μm resin (Dr. Maisch, Ammerbuch-Entringen, Germany), into a self-pulled needle (300-mm length × 75-μm inner diameter). The mobile phases consisted of buffer A (0.1% formic acid and 2% acetonitrile) and B (0.1% formic acid and 90% acetonitrile). Digested peptides were dissolved in a 2% acetonitrile solution containing 0.1% trifluoroacetic acid and loaded onto a trap column (0.075×20 mm, Acclaim PepMap RSLC Nano-Trap Column; Thermo Scientific). The nano-LC gradient was delivered at 280 nL/min and consisted of a linear gradient of buffer B developed from 5–35% B over 180 min. Full MS scans were performed by using an orbitrap mass analyzer (scan range, 350–1800 m/z, with a resolution of 70,000 after accumulation of ions to a 3×10^6^ target value). The twelve most intense precursor ions were selected and fragmented in the octopole collision cell by higher-energy collisional dissociation with a maximum injection time of 120 ms, a normalized collision energy of 30% and a resolution of 35,000. The MS/MS ion-selection threshold was set to 5×10^4^ counts. A 1.0-Da isolation width was chosen.

**The protocol of targeted proteomics**

The digested peptides containing stable isotope-labeled peptide standards were analyzed by using a TSQ-Vantage triple quadruple mass spectrometer (Thermo Fisher Scientific, Bremen, Germany) with a nano-LC interface (AMR, Tokyo, Japan), Paradigm MS2 (Michrom BioResources, Auburn, CA), and an HTC-PAL autosampler (CTC Analytics, Zwingen, Switzerland). The analytical column was packed with reversed-phase material (ReproSil-Pur C18-AQ, 1.9-μm resin; Dr. Maisch, Ammerbuch-Entringen, Germany) into a self-pulled needle (100-mm length × 75-μm inner diameter). The mobile phases consisted of buffer A (0.1% formic acid and 2% acetonitrile) and B (0.1% formic acid and 90% acetonitrile). Digested peptides were dissolved in a 2% acetonitrile solution containing 0.1% trifluoroacetic acid and loaded onto a trap column (0.075 × 20 mm; Acclaim PepMap RSLC Nano-Trap Column; Thermo Scientific). The nano-LC gradient was delivered at 280 nL/min and consisted of a linear gradient of buffer B developed from 5-35% B in 60 min. The parameters of the instrument were set as follows: 0.002 m/z scan width, 0.7 fwhm Q1 resolution, 2.5-s cycle time, and 1.8-mTorr gas pressure. Data acquisition was performed in scheduled SRM mode (time window, 5 min). The settings of the transitions, which indicate pairs of precursor’s *m/z* and product’s *m/z* for monitoring a target peptide in an SRM analysis, were optimized for the twelve target peptides by performing a test run of the synthetic isotope-labeled peptide as previously reported [1]. The transition list is shown in Table 4. The collision energy for each peptide was obtained by the equations, collision energy = 0.034 × precursor m/z - 0.848 for doubly charged precursor ions and collision energy = 0.022 × precursor m/z + 5.953 for triply charged precursor ions.

**Reference**

1. Narumi R, Shimizu Y, Ukai-Tadenuma M, Ode KL, Kanda GN, Shinohara Y, et al. Mass spectrometry-based absolute quantification reveals rhythmic variation of mouse circadian clock proteins. Proc Natl Acad Sci U S A. 2016;113(24):E3461-7.
